# Supplementary material for: Inter-cell type interactions that control JNK signaling in the Drosophila intestine
Source: Nat Commun. 2024 Jun 28;15:5493. doi: 10.1038/s41467-024-49786-w (PMC11214625; doi:10.1038/s41467-024-49786-w)
Supplement: Supplementary file 1 — Supplementary Information [file 41467_2024_49786_MOESM1_ESM.pdf]

Supplementary Information

Inter-cell type interactions that control JNK signaling in the *Drosophila* intestine

Peng Zhang[1,#], Stephen M. Pronovost[1], Marco Marchetti[1], Chenge Zhang[1], Xiaoyu Kang[1], Tahmineh Kandelouei[1], Christopher Li[1,2], and Bruce A. Edgar[1,#]

[1] Huntsman Cancer Institute and Department of Oncological Sciences, University of Utah, Salt Lake City, UT 84112, USA

[2] Harvard University, Cambridge, MA 02138, USA

[#] Correspondence. E-mail: peng.zhang@hci.utah.edu; bruce.edgar@hci.utah.edu

Index:

Supplementary figures S1-S8

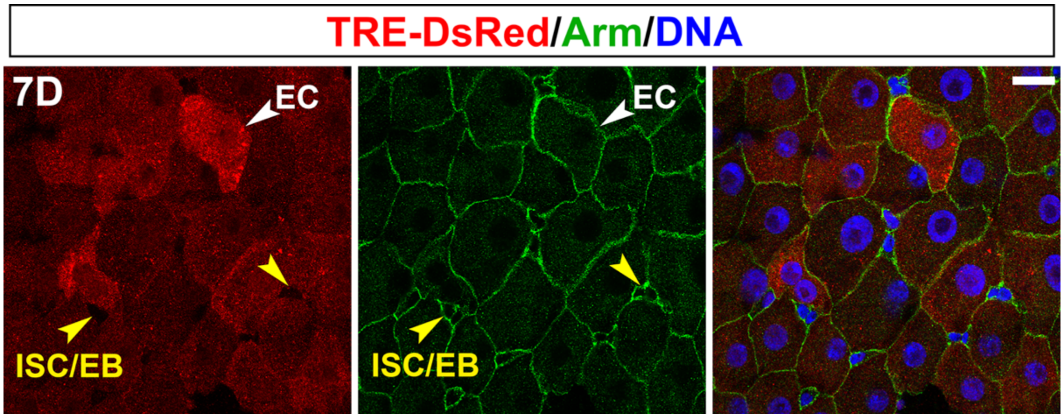

Supplementary Figure 1. The JNK reporter *TRE-DsRed* is exclusively expressed in gut ECs. *TRE-DsRed* flies were raised at 25°C. 7-day-old adult females were dissected. Midguts were stained with anti-Armadillo/-DsRed antibodies. Yellow arrowheads indicate progenitor cells (ISCs/EBs), and white arrowheads indicate ECs. Nuclei were labeled in blue. Images are representative of three independent experiments. Scale bar: 15μm.

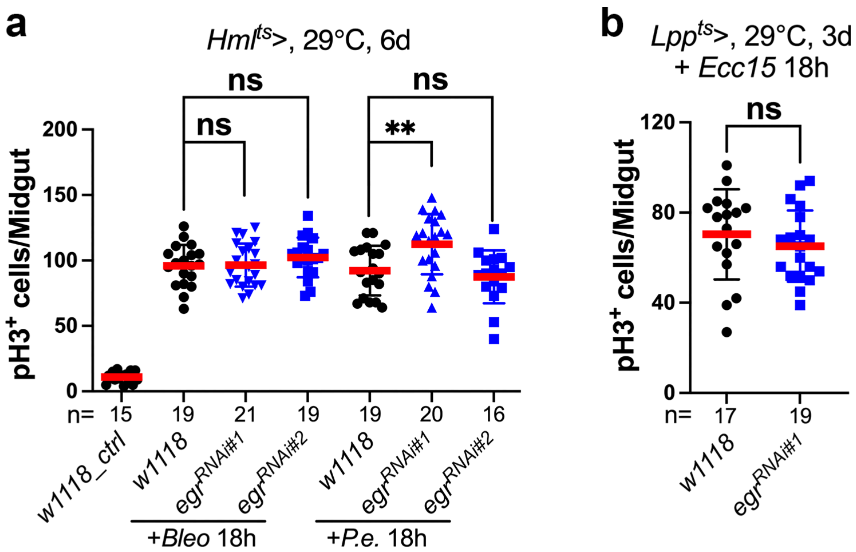

Supplementary Figure 2. Neither hemocyte nor fat body is the source of Eiger for gut damage response. Knockdown of *egr* in hemocyte (a) or fat body (b) was driven by Hml^{ts} or Lpp^{ts}, respectively. 2- to 3-day-old adult females were shifted from 18°C to 29°C for 3 or 6 days (as indicated in panels) before treatments (5% sucrose (control), *P.e.*, 500μM *Bleomycin*, or *Ecc15*) and dissections. Midguts were stained with anti-pH3 antibody, and ISC mitoses were quantified by counting $pH3^+$ cells. Quantification data represent the mean ± SD (two-tailed unpaired *t*-test, $^{ns}P>0.05$, $^{**}P=0.005$). N values in individual panels indicate the number of midguts examined. Source data are provided as a Source Data file.

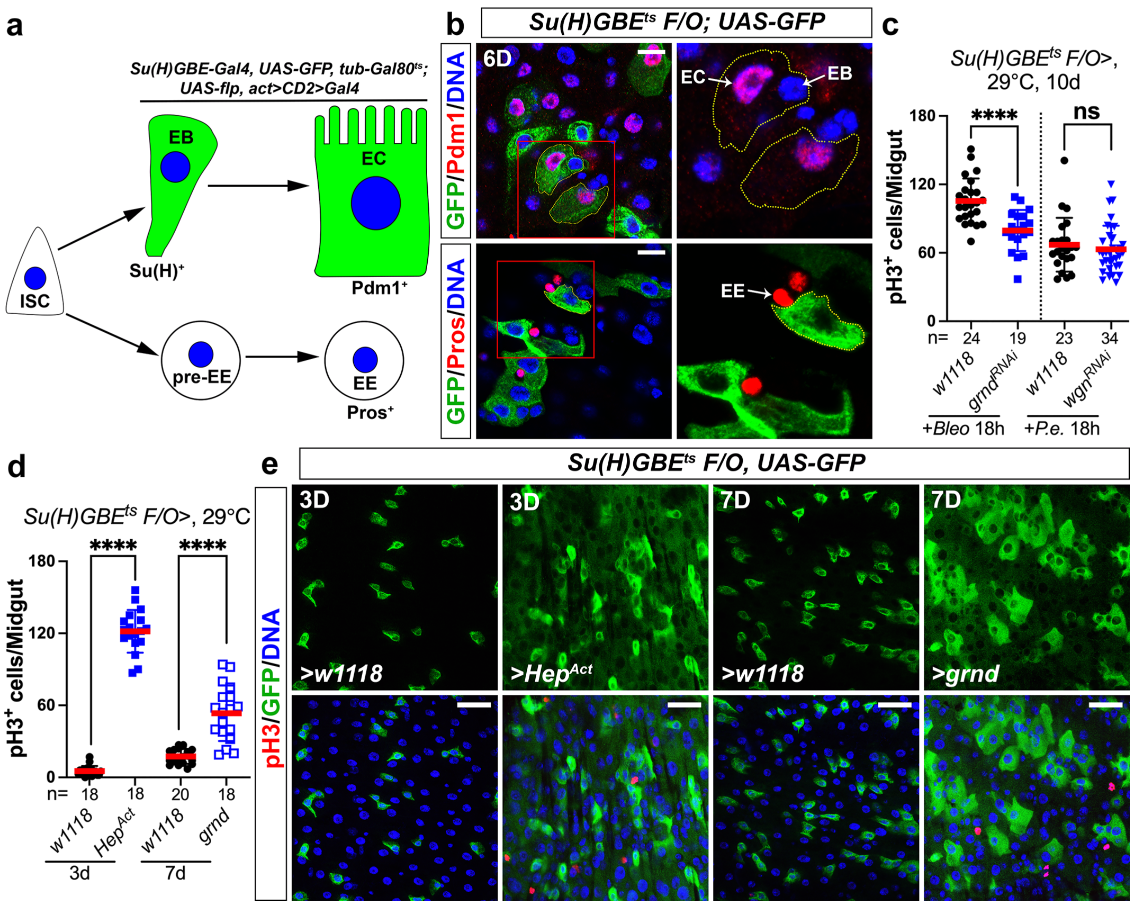

Supplementary Figure 3. Non-autonomous JNK signaling promotes ISC proliferation. (a) Diagram illustrating the "*Su(H)GBE^{ts}-FlipOut*" lineage-tracing system (*Su(H)GBE^{ts} F/O*). EB marker: Suppressor of Hairless (Su(H)); EC marker: Pdm1; EE marker: Prospero (Pros). (b) Validation of the *Su(H)GBE^{ts} F/O* system. 2- to 3-day-old adult females were shifted from 18°C to 29°C for 6 days. Midguts were stained with anti-GFP/-Pdm1/-Pros antibodies, with nuclei labeled in blue. The red boxes in the left panels were enlarged in the right panels. In this system, EBs (Pdm1^{-}, small nuclei) are labeled with GFP following a temperature shift, and then all newborn ECs (Pdm1^{+}) inherit GFP expression. No EE (Pros^{+}) was observed in the GFP^{+} clones, confirming that EEs are directly derived from ISCs rather than EBs. (c-e) Midguts were stained with anti-GFP/-pH3 antibodies, with nuclei labeled in blue. (c, d) ISC mitoses were quantified by counting pH3^{+} cells. Quantification data represent the mean ± SD (two-tailed unpaired *t*-test, ^{ns}P=0.4961, ****P<0.0001). N values in individual panels indicate the number of midguts examined. (c) Knockdown of *grnd* or *wgn* in the EB-EC lineage was driven by *Su(H)GBE^{ts} F/O* at 29°C for 10 days before treatments (*P.e.* or 250µM *Bleomycin*). (d-e) *Hep^{Act}* or *grnd* was overexpressed using *Su(H)GBE^{ts} F/O* at 29°C for 3 or 7 days. High mitoses (d) and nearly complete epithelial replacement (e) are observed in *Hep^{Act}*- or *grnd*-overexpressing guts. Images in (b) are representative of three independent experiments; (e), two independent experiments. Scale bars: 10µm in (b) and 30µm in (e). Source data are provided as a Source Data file.

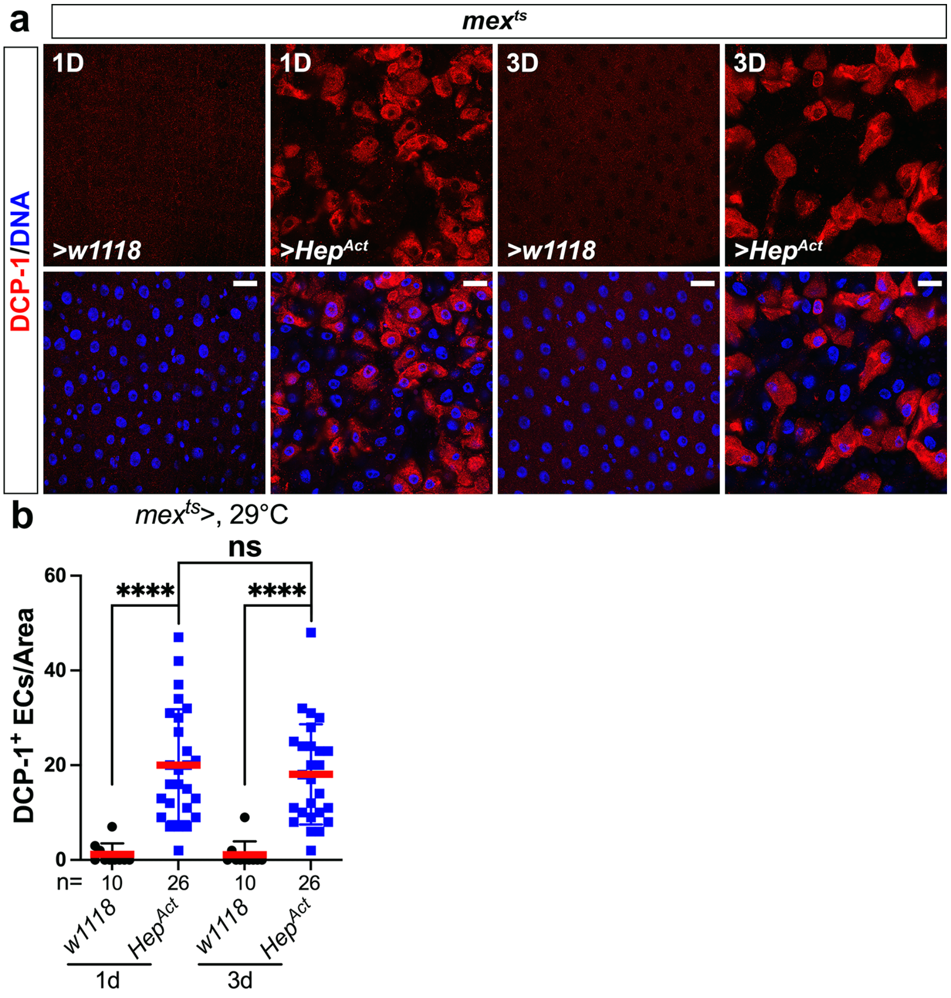

Supplementary Figure 4. Hyperactivation of JNK triggers EC apoptosis in a dose-independent manner. (a) *UAS-Hep^Act^* was overexpressed in progenitor cells driven by mex^{ts}. mex^{ts}*>w1118* flies were used as controls. Flies were raised at 18°C and then shifted to 29°C for 1 day or 3 days before dissection. Midguts were stained with the anti-DCP-1 (apoptotic marker) antibody. Nuclei were labeled in blue. Images are representative of three independent experiments. Scale bars: 20μm. (b) the degree of EC apoptosis (DCP-1^{+} ECs/Area) was quantified. Quantification data represent the mean ± SD (two-tailed unpaired *t*-test, ^{ns}P=0.5392, ****P<0.0001). N values indicate the number of imaging areas examined. Source data are provided as a Source Data file.

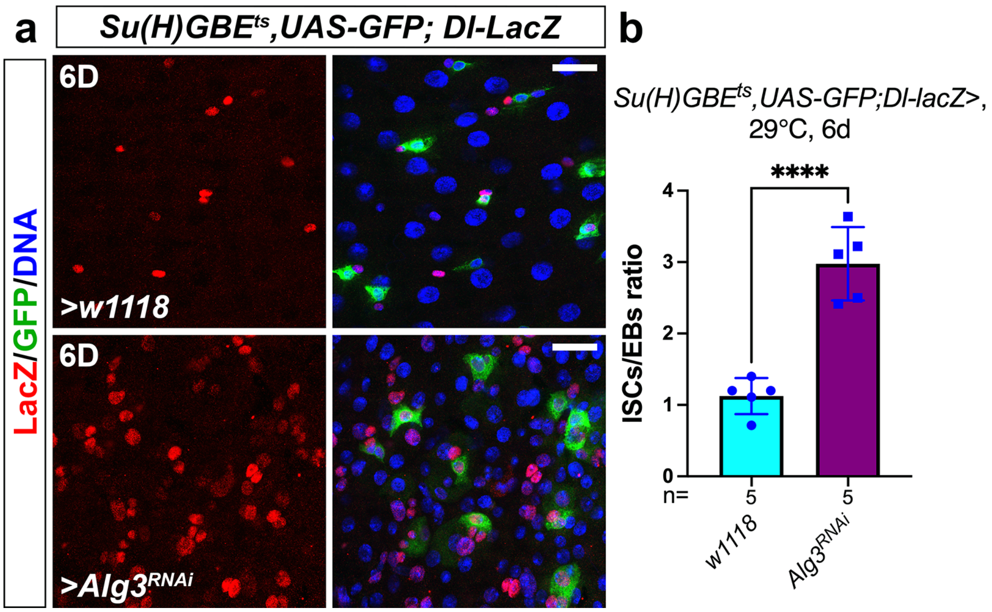

Supplementary Figure 5. The cell non-autonomous role of ALG3 in restraining intestinal stem cell pool size. (a-b) Knockdown of *Alg3* in EBs was driven by *Su(H)GBE^{ts}* at 29°C for 6 days. *Su(H)GBE^{ts}>w1118* flies were used as controls. Midguts were stained with anti-GFP/-LacZ antibodies. *Dl-lacZ* was used to label ISCs. Nuclei were labeled in blue. Images are representative of three independent experiments. Scale bars: 20μm. (b) Quantification of ISCs/EBs ratio: control (n = 5 areas) versus *Alg3^{RNAi}* (n = 5 areas) (mean ± SD, two-tailed unpaired *t*-test, ****P < 0.0001). Source data are provided as a Source Data file.

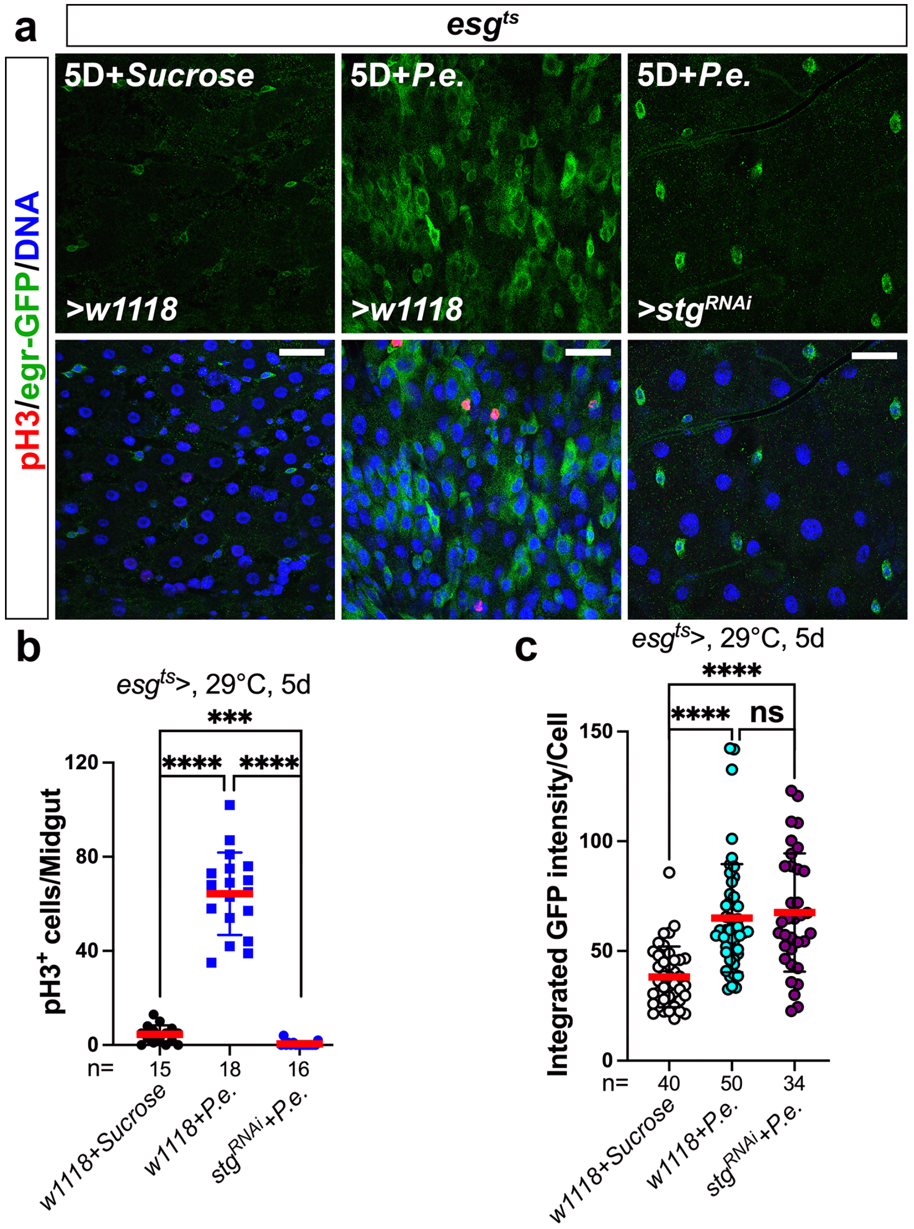

Supplementary Figure 6. Stress-induced *eiger* upregulation is not a secondary consequence of ISC hyperplasia. (a) *esg*^{ts}*>egr-GFP* and *esg*^{ts}*>egr-GFP;stg*^{RNAi} female flies were shifted from 18°C to 29°C for 5 days before treatments (5% sucrose or *P.e.* for 18h) and dissections. Midguts were stained with anti-GFP/-pH3 antibodies, with nuclei labeled in blue. Images are representative of three independent experiments. Scale bars: 30μm. (b) ISC mitoses were quantified by counting pH3^{+} cells. N values indicate the number of midguts examined. (c) Integrated GFP intensity per progenitor of each genotype was quantified and compared. N values indicate the number of cells examined. Quantification data represent the mean ± SD (two-tailed unpaired *t*-test, ^{ns}P=0.6544, ***P=0.0004, and ****P<0.0001). Source data are provided as a Source Data file.

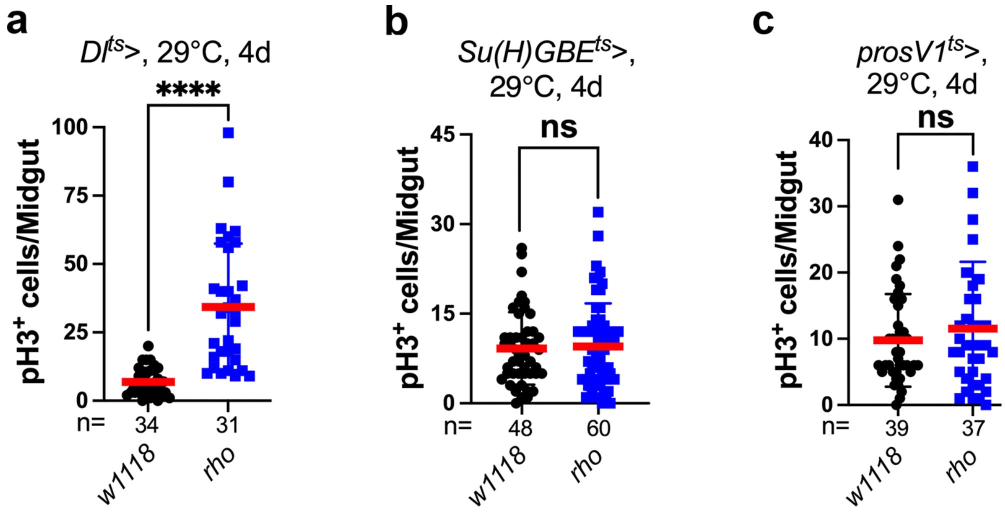

Supplementary Figure 7. Pro-mitotic effects of Rhomboid in individual gut cell types. Overexpression of *Rho* was conducted in individual gut cell types using specific drivers: *Dl*^{ts} (a, ISC-driver), *Su(H)GBE*^{ts} (b, EB-driver), and *ProsV1*^{ts} (c, EE-driver). Flies were raised at 18°C. 2- to 3-day-old adult females were shifted from 18°C to 29°C for 4 days before dissections. Midguts were stained with anti-pH3 antibody, and ISC mitoses were quantified by counting pH3$^+$ cells. Quantification data represent the mean ± SD (two-tailed unpaired *t*-test, ^{ns}P=0.8115 (b), ^{ns}P=0.3738 (c), ****P<0.0001). N values in individual panels indicate the number of midguts examined. Source data are provided as a Source Data file.

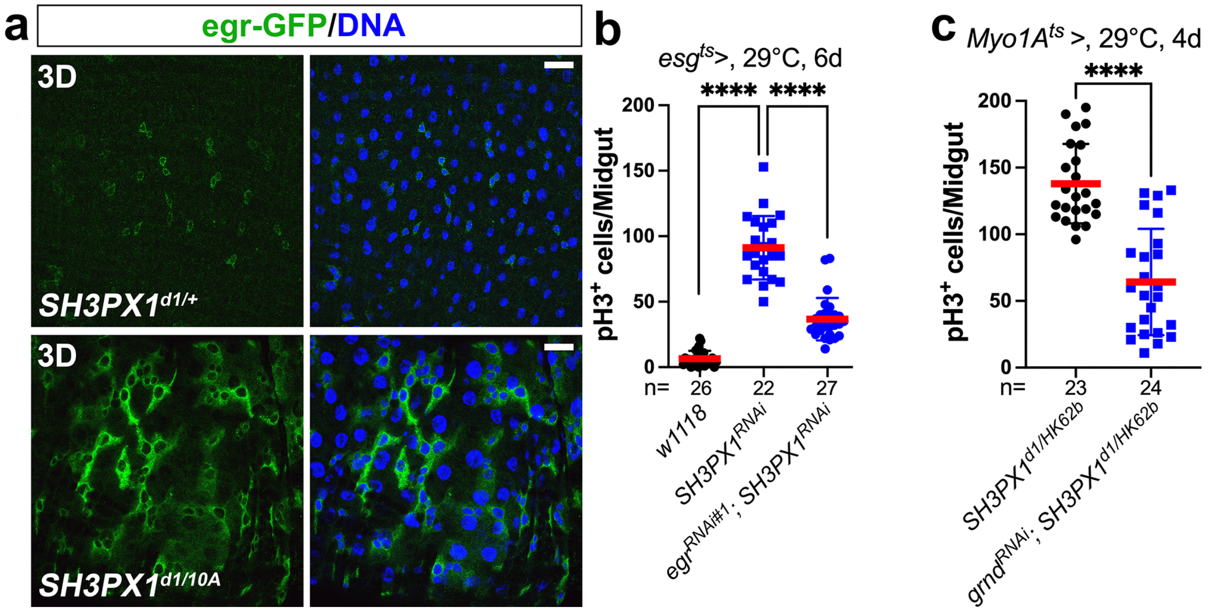

Supplementary Figure 8. *Eiger* is a downstream target of SH3PX1. (a) Flies were raised at 25°C. 3-day-old *egr-GFP/+;SH3PX1*$^{d1/+}$ and *egr-GFP/+;SH3PX1*$^{d1/10A}$ adult females were dissected. Midguts were stained with anti-GFP antibody. Nuclei were labeled in blue. Images are representative of three independent experiments. Scale bars: 20µm. (b) *w1118* (control), *SH3PX1*^{RNAi}, or *egr*^{RNAi}+*SH3PX1*^{RNAi} was overexpressed using *esg*^{ts} at 29°C for 6 days. (c) *UAS-grnd*^{RNAi} was overexpressed using *Myo1A*^{ts} at 29°C for 4 days in the trans-heterozygous *SH3PX1*$^{d1/HK62b}$ mutant background. *SH3PX1*$^{d1/HK62b}$ flies were selected as controls. (b-c) Midguts were stained with anti-pH3 antibody, and ISC mitoses were quantified by counting pH3^{+} cells. Quantification data represent the mean ± SD (two-tailed unpaired *t*-test, ****P<0.0001). N values in individual panels indicate the number of midguts examined. Source data are provided as a Source Data file.
