# Supplementary material for: Inter-cell type interactions that control JNK signaling in the Drosophila intestine
Source: Nat Commun. 2024 Jun 28;15:5493. doi: 10.1038/s41467-024-49786-w (PMC11214625; doi:10.1038/s41467-024-49786-w)
Supplement: Supplementary file 3 — Reporting Summary [file 41467_2024_49786_MOESM3_ESM.pdf]

nature portfolio

Corresponding author(s): Bruce A. Edgar
Peng Zhang

Last updated by author(s): Jun 1, 2024

Reporting Summary

Nature Portfolio wishes to improve the reproducibility of the work that we publish. This form provides structure for consistency and transparency in reporting. For further information on Nature Portfolio policies, see our Editorial Policies and the Editorial Policy Checklist.

Statistics

For all statistical analyses, confirm that the following items are present in the figure legend, table legend, main text, or Methods section.

n/a	Confirmed	
☐	☒	The exact sample size (n) for each experimental group/condition, given as a discrete number and unit of measurement
☐	☒	A statement on whether measurements were taken from distinct samples or whether the same sample was measured repeatedly
☐	☒	The statistical test(s) used AND whether they are one- or two-sided<br>*Only common tests should be described solely by name; describe more complex techniques in the Methods section.*
☒	☐	A description of all covariates tested
☒	☐	A description of any assumptions or corrections, such as tests of normality and adjustment for multiple comparisons
☐	☒	A full description of the statistical parameters including central tendency (e.g. means) or other basic estimates (e.g. regression coefficient) AND variation (e.g. standard deviation) or associated estimates of uncertainty (e.g. confidence intervals)
☐	☒	For null hypothesis testing, the test statistic (e.g. F, t, r) with confidence intervals, effect sizes, degrees of freedom and P value noted<br>*Give P values as exact values whenever suitable.*
☒	☐	For Bayesian analysis, information on the choice of priors and Markov chain Monte Carlo settings
☒	☐	For hierarchical and complex designs, identification of the appropriate level for tests and full reporting of outcomes
☒	☐	Estimates of effect sizes (e.g. Cohen's d, Pearson's r), indicating how they were calculated

Our web collection on statistics for biologists contains articles on many of the points above.

Software and code

Policy information about availability of computer code

Data collection	Confocal images were acquired using a Zeiss LSM 780 or Leica SP8 confocal microscope. qPCR data was collected on the BioRad CFX384TM Real-Time System.
Data analysis	All statistical analyses were conducted using GraphPad Prism 10. Quantitative imaging data were analyzed using Fiji (ImageJ). qPCR data were analyzed on the Bio-Rad CFX384TM Real-Time System using CFX Manager software (v3.1).

For manuscripts utilizing custom algorithms or software that are central to the research but not yet described in published literature, software must be made available to editors and reviewers. We strongly encourage code deposition in a community repository (e.g. GitHub). See the Nature Portfolio guidelines for submitting code & software for further information.

Data

Policy information about availability of data

All manuscripts must include a data availability statement. This statement should provide the following information, where applicable:

- Accession codes, unique identifiers, or web links for publicly available datasets
- A description of any restrictions on data availability
- For clinical datasets or third party data, please ensure that the statement adheres to our policy

All data generated in this study are provided in the main figures, supplementary information, and the source data file. The raw confocal microscopy images supporting the findings of this study are available from the corresponding authors upon request. Source data are provided with this paper.

Research involving human participants, their data, or biological material

Policy information about studies with human participants or human data. See also policy information about sex, gender (identity/presentation), and sexual orientation and race, ethnicity and racism.

Reporting on sex and gender	N/A
Reporting on race, ethnicity, or other socially relevant groupings	N/A
Population characteristics	N/A
Recruitment	N/A
Ethics oversight	N/A

Note that full information on the approval of the study protocol must also be provided in the manuscript.

Field-specific reporting

Please select the one below that is the best fit for your research. If you are not sure, read the appropriate sections before making your selection.

☒ Life sciences ☐ Behavioural & social sciences ☐ Ecological, evolutionary & environmental sciences

For a reference copy of the document with all sections, see nature.com/documents/nr-reporting-summary-flat.pdf

Life sciences study design

All studies must disclose on these points even when the disclosure is negative.

Sample size	No statistical methods were used to predetermine sample size. Sample sizes for experiments were consistent with those commonly used in the field, typically ranging from 5 to 20 midguts per experiment.
Data exclusions	No data points were excluded from analysis.
Replication	Each experiment was replicated two or more times, unless otherwise indicated.
Randomization	Animals were selected based on their genotype and then randomly assigned for use in experiments.
Blinding	No blinding was performed in this study, and techniques were applied uniformly to all samples in parallel.

Reporting for specific materials, systems and methods

We require information from authors about some types of materials, experimental systems and methods used in many studies. Here, indicate whether each material, system or method listed is relevant to your study. If you are not sure if a list item applies to your research, read the appropriate section before selecting a response.

Materials & experimental systems

n/a	Involved in the study	
☐	☒	Antibodies
☒	☐	Eukaryotic cell lines
☒	☐	Palaeontology and archaeology
☐	☒	Animals and other organisms
☒	☐	Clinical data
☒	☐	Dual use research of concern
☒	☐	Plants

Methods

n/a	Involved in the study	
☒	☐	ChIP-seq
☐	☒	Flow cytometry
☒	☐	MRI-based neuroimaging

Antibodies

Antibodies used	The following primary antibodies (including dilutions) were used for Immunostaining: chicken α-GFP (Thermo Fisher Scientific, #A10262, 1:1000), rabbit α-pH3 (Millipore, # 06-570, 1:1000), mouse α-β-galactosidase (Promega, #Z3781, 1:300), rabbit α-Cleaved Drosophila Dcp-1 (Asp215) (Cell Signaling Technology, #9578, 1:100), rabbit α-Pdm1 (gift from X. Yang at Zhejiang University, China, 1:200), rat α-HA (Roche, # 11867423001, 1:300), rabbit α-DsRed (Takara Bio Clontech, #632496, 1:400), mouse α-Armadillo (DSHB, #N2 7A1, 1:10). Staining signals were detected using species-appropriate secondary antibodies conjugated with Alexa Fluor 488 and/ or 568. (Thermo Fisher Scientific, 1:1000). Nuclei were labelled using DAPI (Thermo Fisher Scientific, #D1306, 1:1000) or Hoechst

	33342 (Thermo Fisher Scientific, #62249, 1:1000). The following antibodies are used for western blot: mouse α-Tubulin (DSHB, #12G10, 1:250) and mouse α-V5 (Invitrogen, #46-0705, 1:2000).
Validation	All antibodies used in this study have been validated either by the manufacturers or through our previous research and studies from other laboratories.

Animals and other research organisms

Policy information about studies involving animals; ARRIVE guidelines recommended for reporting animal research, and Sex and Gender in Research

Laboratory animals	Drosophila melanogaster, adult females
Wild animals	N/A
Reporting on sex	N/A
Field-collected samples	N/A
Ethics oversight	N/A

Note that full information on the approval of the study protocol must also be provided in the manuscript.

Plants

Seed stocks	N/A
Novel plant genotypes	N/A
Authentication	N/A

Flow Cytometry

Plots

Confirm that:

- ☐ The axis labels state the marker and fluorochrome used (e.g. CD4-FITC).
- ☐ The axis scales are clearly visible. Include numbers along axes only for bottom left plot of group (a 'group' is an analysis of identical markers).
- ☐ All plots are contour plots with outliers or pseudocolor plots.
- ☐ A numerical value for number of cells or percentage (with statistics) is provided.

Methodology

Sample preparation	Midguts were dissected in cold nuclease-free PBS and subsequently digested with 5mg/mL Collagenase for 1 hour at 29°C in the thermomixer (850 rpm). Samples were pipetted using a cut tip every 15 mins to help dissociation. Following dissociation, samples were centrifuged at 600g for 10min, resuspended in cold nuclease-free PBS, and filtered into a 35um strainer cap FACS tube.
Instrument	BD FACSAria cell sorter
Software	BD FACSDiva™ software version 8.0
Cell population abundance	Approximately 20,000 GFP positive single progenitors per sample were collected for subsequent RT-qPCR analysis.
Gating strategy	First, the FSC gate was used to select cell particles. Then, the GFP+/PI- gate was used to select live cells. Finally, the single-cell gate (FSC-A/FSC-W) was applied to isolate single cells.

☐ Tick this box to confirm that a figure exemplifying the gating strategy is provided in the Supplementary Information.
